# Influenza and Pneumococcal Vaccination and the Risk of COVID-19: A Systematic Review and Meta-Analysis

**DOI:** 10.3390/diagnostics12123086

**Published:** 2022-12-07

**Authors:** Georgia V. Kapoula, Konstantina E. Vennou, Pantelis G. Bagos

**Affiliations:** 1 Department of Biochemistry, General Hospital of Lamia, 35131 Lamia, Greece; 2 Department of Computer Science and Biomedical Informatics, University of Thessaly, 35131 Lamia, Greece

**Keywords:** influenza vaccine, pneumococcal vaccine, SARS-CoV-2, systematic review, meta-analysis

## Abstract

A number of studies have investigated the potential on-specific effects of some routinely administered vaccines (e.g., influenza, pneumococcal) on COVID-19 related outcomes, with contrasting results. In order to elucidate this discrepancy, we conducted a systematic review and meta-analysis to assess the association between seasonal influenza vaccination and pneumococcal vaccination with SARS-CoV-2 infection and its clinical outcomes. PubMed and medRxiv databases were searched up to April 2022. A random effects model was used in the meta-analysis to pool odds ratio (OR) and adjusted estimates with 95% confidence intervals (CIs). Heterogeneity was quantitatively assessed using the Cochran’s *Q* and the *I*^2^ index. Subgroup analysis, sensitivity analysis and assessment of publication bias were performed for all outcomes. In total, 38 observational studies were included in the meta-analysis and there was substantial heterogeneity. Influenza and pneumococcal vaccination were associated with lower risk of SARS-CoV-2 infection (OR: 0.80, 95% CI: 0.75–0.86 and OR: 0.70, 95% CI: 0.57–0.88, respectively). Regarding influenza vaccination, it seems that the majority of studies did not properly adjust for all potential confounders, so when the analysis was limited to studies that adjusted for age, gender, comorbidities and socioeconomic indices, the association diminished. This is not the case regarding pneumococcal vaccination, for which even after adjustment for such factors the association persisted. Regarding harder endpoints such as ICU admission and death, current data do not support the association. Possible explanations are discussed, including trained immunity, inadequate matching for socioeconomic indices and possible coinfection.

## 1. Introduction

In 2020 in Wuhan, China, cases of pneumonia were reported [1], caused by SARS-CoV-2, which is a coronavirus (CoV), and which lead to a pandemic outbreak. The coronaviruses belong in the *Nidovirales* order and in the *Coronaviridae* family, which has four genera divisions, the alpha, beta, gamma, and delta coronaviruses. All coronaviruses contain very large genomes, are enveloped, and are non-segmented positive-sense ribonucleic acid (RNA) viruses [2]. Coronaviruses in humans mainly cause respiratory diseases, and there are many viruses in this family, with a large spectrum of diseases from the less-severe common cold to serious and highly fatal diseases such as the Severe Acute Respiratory Syndrome (SARS) and the Middle East Respiratory Syndrome (MERS). SARS-CoV-2 is a betacoronavirus in the same subgenus as SARS-CoV-2 and shares RNA with it as well as with the MERS-CoV [3]. The novel coronavirus that we are facing can cause severe disease, a syndrome termed COVID-19. As of 1 April 2022 there are a total of 486,761,597 confirmed cases and 6,142,735 confirmed deaths worldwide [4].

Seasonal influenza is another acute respiratory infection caused by the influenza viruses. Influenza viruses are part of the *Orthomyxoviridae* family, in which there are three main genera: A, B and C [5]. They are RNA viruses with segmented, negative-strand genomes. The diseases caused by influenza range from mild to more severe and even include death in high-risk groups [6]. As one can see, both diseases are respiratory viral diseases, they co-exist at the same period, and they can be clinically indistinguishable (fever, cough, nasal congestion or rhinorrhea, myalgia etc.) [7].

Another respiratory infection is pneumonia. The main causes of pneumonia are viruses, bacteria and fungi. One of the most common bacteria which cause pneumonia (mainly in children) is *Streptococcus pneumonia* [8]. Some studies have shown the co-infection of COVID-19 and influenza or COVID-19 and pneumonia [9].

One step taken towards the end of the pandemic was vaccination. There are ten vaccines that have been approved by the World Health Organization (WHO) [10]. Many non-SARS-CoV-2 vaccines have been tested for preventing SARS-CoV-2 infection or having a protective effect with regard to the severity of the disease. The booster Bacillus Calmette-Guérin (BCG) vaccine was suggested, by many studies and even by a meta-analysis, to have beneficial effects on preventing COVID-19 infection [11,12,13]. Some preliminary studies suggest some protection against SARS-CoV-2 to be conferred from vaccination to other pathogens such as influenza [14], while others have produced contrasting results [15]. Additionally, some researchers suggest that influenza vaccination would help to lighten the burden on health systems and would decrease the transmission probability of COVID-19 [16]. Moreover, some studies have shown a bacteria-virus synergy that could potentially influence the COVID-19 related outcomes [17]. Finally, Venkatakrishnan and coworkers went even further, suggesting that the mutational profile of the SARS-CoV-2 Omicron variation could have been acquired by pattern switching between the SARS-CoV-2 and the viruses that coinfect the same host cells [18]. Due to the aforementioned reasons, we conducted a systematic review and meta-analysis to assess the overall association between influenza and pneumococcal vaccination and SARS-CoV-2 infection and to investigate the potential factors that influence the contradictory results.

## 2. Materials and Methods

A comprehensive search was performed to identify papers that investigated influenza and pneumococcal vaccination and SARS-CoV-2 infection and severity of the disease. PubMed [19] and medRxiv [20] were investigated for relevant research articles.

The search terms for influenza vaccination were: (“Influenza Vaccines” OR “influenza vaccine” OR “flu vaccine” OR “anti-flu vaccine”) AND (“SARS-CoV-2” OR “covid-19” OR “2019-ncov” OR “2019nconv” OR “sars-cov2” OR “COVID-19”) in PubMed and “influenza vaccine” (abstract or title, match all words) in medRxiv. For pneumococcal vaccination, the terms used were: (“pneumococcal Vaccines” OR “pneumococcal vaccine”) AND (“SARS-CoV-2” OR “COVID-19” OR “2019-ncov” OR “2019nconv” OR “sars-cov2” OR “COVID-19”) in PubMed and “pneumococcal vaccine” (abstract or title, match all words) in medRxiv. To avoid selection bias, no filter was applied in language, year, method, etc. This systematic review was performed according to Preferred Reporting Items for Systematic Reviews and Meta-Analyses (PRISMA) guidelines [21], the STROBE Statement checklist [22], and other general guidelines [23]. The search was performed up to 30 April 2022.

The information that was extracted from the selected studies was author, title, year of publication, country in which it was conducted, study design, study population, sample size, gender, comorbidities, COVID-19 outcome, the effect size related to relevant outcomes (e.g., OR, HR, RR), and other potentially relevant information. Since the risk for COVID-19 varies largely according to risk factors, studies not including adjusted odds ratio (OR), adjusted hazard ratio (HR) or adjusted relative risk (RR) as their effect size were excluded. We requested that the effect sizes were adjusted mainly for gender, age, race, comorbidities, smoking status, etc.

The main inclusion criteria were (a) vaccination period for influenza or pneumococcal from autumn 2019 until autumn 2021; (b) confirmed COVID-19 infection with either real-time PCR or serologic test, or according to WHO definition or laboratory confirmation (not specified); (c) participants who were adults; and (d) availability of an adjusted estimate of OR, HR or RR (see above).

In order to assess the association between the two vaccines (influenza and pneumococcal) and COVID-19 outcome (infection, death, hospitalization, etc.), a random effects meta-analysis was performed for each outcome, using the adjusted OR, or adjusted RR/HR, combined with their 95% confidence interval (CI) as effect size [24].The between studies heterogeneity of the pooled estimates was quantified by means of the chi-square-based Cochran’s *Q* statistic and the consistency index (*I*^2^) [25]. To assess publication bias, the rank correlation method of Begg and Mazumdar [26], and the fixed effects regression method of Egger [27] were applied. Subgroup analyses were performed to investigate the effect of various study-level characteristics. Sensitivity analyses were performed by omitting one study at a time to assess studies with a notable impact and examine the robustness of the overall effect.

For all statistical analyses performed, the statistical software package STATA 13 [28] was used and results with *p*-value < 0.05 were considered statistically significant.

## 3. Results

### 3.1. Study Selection and Study Characteristics

**Figure 1:**
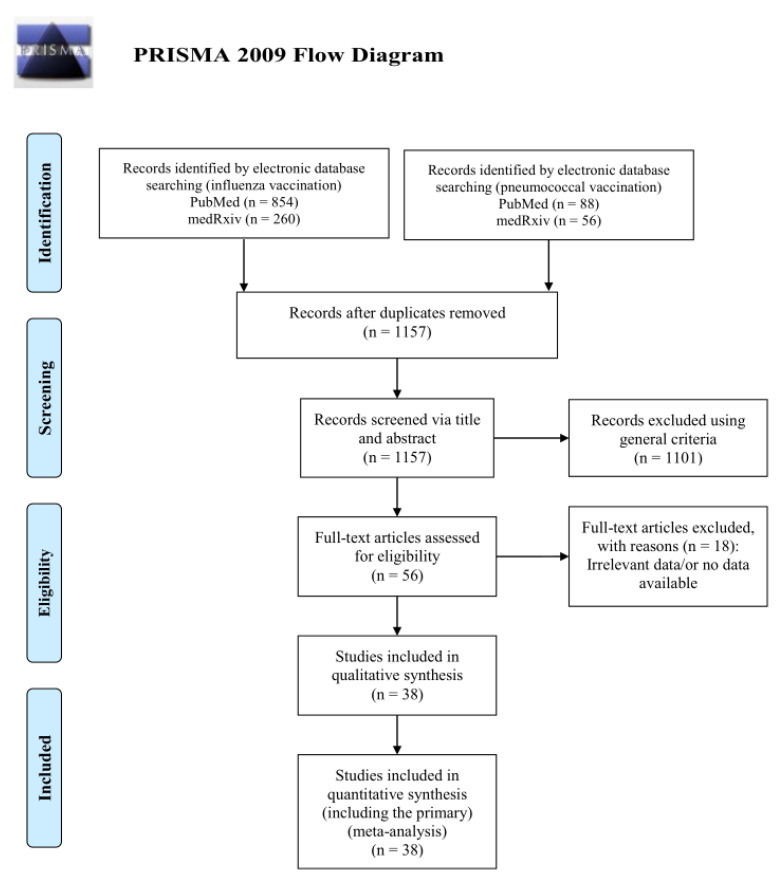
The PRISMA flow diagram for the included studies.

The literature search from the two databases identified a total of 1114 studies related to the influenza vaccination and 144 records for the pneumococcal vaccination search. After reading the titles and excluding duplicates, 1020 and 137 unique publications remained for influenza and pneumococcal vaccination, respectively. The simultaneous titles and abstracts review led to a further rejection of 1101 publications. The remaining 56 articles were read in full copy, as well as the citations of the retrieved articles. Finally, 38 publications met all the inclusion criteria for the quantitative analysis and were included in the final systematic review and meta-analysis. The PRISMA flow diagram of the study selection procedure is shown in Figure 1.

Among the included publications, 22 studies focused on the association between influenza vaccination and the risk of SARS-CoV-2 infection and 10 studies on the association between pneumococcal vaccination and the risk of SARS-CoV-2 infection. These studies included a total of 55,917,587 individuals (COVID-19 patients and non-COVID patients).

Out of the 38 publications included in the meta-analysis, 19 publications assessed the association of the influenza vaccination and COVID-19 clinical outcomes. The different outcomes included: (a) need for hospitalization involving 15 studies with 55,881,321 individuals (13,123,605 vaccinated and 42,758,116 unvaccinated patients); (b) the administration of mechanical ventilation/invasive respiratory oxygen support described in four studies encompassing 255,735 patients; (c) intensive care unit admission. This outcome was assessed in 11 studies involving 446,924 vaccinated and not vaccinated patients and (d) mortality, which was assessed in 18 studies involving 407,409 vaccinated and not vaccinated COVID-19 patients. Moreover, four studies assessed the association between pneumococcal vaccination and hospitalization, including a total of 4310 patients, of which 1333 were vaccinated and 2977 were not vaccinated. Finally, three studies examined the association between pneumococcal vaccination and intensive care unit admission involving 1231 patients (921 non-vaccinated Vs 310 vaccinated). The baseline characteristics of the included studies are listed in Table 1 and Table S1 (influenza vaccination and SARS-CoV-2 infection), Table 2 and Table S2 (influenza vaccination and clinical outcomes), and Table 3 and Table S3 (pneumococcal vaccination and SARS-CoV-2 infection and clinical outcomes). Most studies enrolled individuals from the general population. Among them, five studies had health workers as a population study [29,30,31,32,33], one study involved only pregnant women [34], another study included only transplant recipients [35], and four studies were performed in older adults (age > 65) [36,37,38,39]. The laboratory method (rt-PCR) used to diagnose the disease was common in most of the included studies. The adjustment variables varied across studies with age, gender and comorbidities being the most common ones, reflecting the known COVID-19 risk factors. Studies which did not contain adjusted estimates (OR or RR/HR) as well as ecological studies were excluded from the meta-analysis and are summarized in Table S4.

### 3.2. The Association between Influenza Vaccination and SARS-CoV-2 Infection

**Figure 2:**
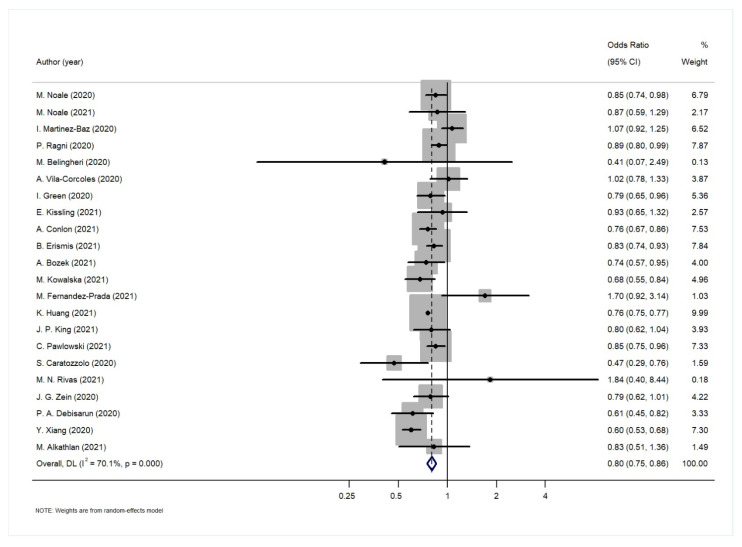
Forest plot for the association between influenza vaccination and SARS-Cov-2 infection.

The results of the random effects analysis are summarized in Table 4. The association of the influenza vaccination and SARS-CoV-2 infection is presented graphically in the forest plot in Figure 2. Influenza vaccination is shown to be associated with lower risk of SARS-CoV-2 infection (random effects model: pooled estimate 0.80, 95% CI: 0.75–0.86). The results of the statistical tests for publication bias (Begg’s, Egger’s test) are shown in Table 4. Both tests showed no evidence of publication bias for the pooled estimates of the association between influenza vaccination and SARS-CoV-19 infection (*p*-value > 0.05). In the leave-one-out sensitivity analyses regarding the association between influenza vaccination and SARS-CoV-2 infection, the results showed that no individual study influenced the overall effect estimate.

The between-studies heterogeneity was substantial with *I*^2^ = 70.1%. In order to investigate the high degree of heterogeneity, we performed a subgroup analysis investigating the potential effect of study-level characteristics (study design, study population, effect size, adjustment factors, method for assessment of COVID-19, and continent). However, none of these factors seemed to play an important role, since in all cases the results of the subgroup analysis are quite close to the overall estimate (Table 5). It is worth noting, however, that the association was diminished when the analysis was restricted to the six studies that adjusted for age, gender, comorbidities and some indices of socioeconomic status (such as residence, income or education).

Moreover, two studies (Debisarum et al. [50], Erismis et al. [33]) were scrutinized further, as in their analysis they used only age and gender as adjustment variables. However, when both were excluded from the meta-analysis and the overall meta-analysis was repeated, the results did not differ from the overall estimate: OR 0.81 (95% CI: 0.75–0.87).

### 3.3. The Association between Influenza Vaccination and COVID19 Clinical Outcomes

**Figure 3:**
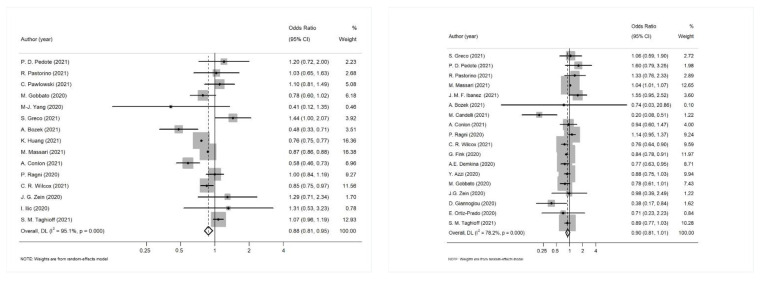
(**Left**): Forest plot for the association between influenza vaccination and COVID-19 related hospitalization; (**Right**): Forest plot for the association between influenza vaccination and mortality.

The results of the meta-analysis that was conducted separately for each clinical outcome of interest are shown in Table 4. The results suggest a potential association between influenza vaccination and hospitalization (OR: 0.88, 95% CI: 0.81–0.96) and mechanical ventilation/invasive respiratory support (OR: 0.73, 95% CI: 0.58–0.92), but not with ICU admission (OR: 0.96, 95% CI: 0.88–1.06) and mortality (OR: 0.90, 95% CI: 0.81–1.01). Both Begg’s and Egger’s tests showed no evidence of publication bias for the pooled estimates of the association between influenza vaccination and COVID-19 related clinical outcomes (all *p*-values > 0.05). The summary of the publication bias tests is presented in Table 4. Figure 3 represents the forest plot for the association between influenza vaccination and COVID-19 related hospitalization and between influenza vaccination and mortality.

In the sensitivity analyses that followed, the pooled estimates were consistent when any single study was omitted. A subgroup analysis was performed to explain the between studies heterogeneity for the association of influenza vaccination and COVID-19 clinical outcomes (Table 6). Study design was not used as a variable in this subgroup analysis, as the majority of the studies were cohort studies with the exception of one cross-sectional study in the hospitalization outcome and two cross-sectional study investigating the mortality outcome. Study population was also not used, as the majority of the studies involved adults from the general population. Moreover, due to the limited studies reporting adjusted measures of the effect of the association between influenza vaccination and the need for mechanical ventilation, a subgroup analysis could not be performed in this case.

### 3.4. The Association between Pneumococcal Vaccination with SARS-CoV-2 Infection and Its Clinical Outcomes

**Figure 4:**
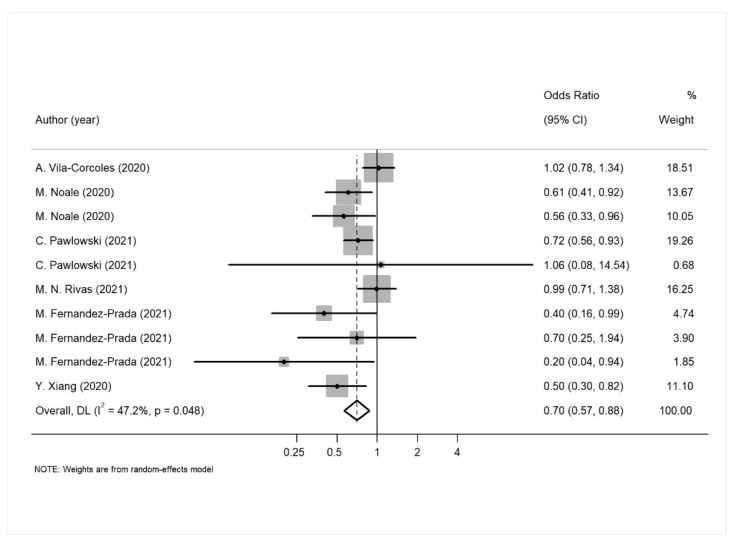
Forest plot for the association between pneumococcal vaccination and SARS-CoV-2 infection.

A meta-analysis of the association between pneumococcal vaccination with SARS CoV-2 infection, the need of hospitalization, and ICU admission was performed. As a result, pneumococcal vaccination was shown to be associated with a lower risk of COVID-19 infection (Figure 4, Table 7). The majority of the studies here (seven out of 10) provided estimates adjusted for age, gender, comorbidities and some indices of socioeconomic status (such as residence, income or education). The overall effect did not change, even when we restricted the analysis in the sample (OR: 0.61, 95% CI: 0.44–0.85).

The results of the meta-analysis regarding pneumococcal vaccination and the need for hospitalization showed that the vaccine seems to be associated with a higher risk of COVID-19 hospitalization. However, only four studies investigated the particular association, and additionally there was evidence for publication bias in this analysis, making the results questionable. Finally, pneumococcal vaccination was not associated with the need of intensive care, but as before, only three studies investigated this association.

## 4. Discussion

Influenza and COVID-19 are different respiratory viral diseases that can be clinically indistinguishable and that can co-exist in the same period. Some preliminary studies suggest some protection against SARS-CoV-2 to be conferred from vaccination for other pathogens such as influenza [14], while others have produced a contrasting result [15]. There is also a limited number of studies that examined the association of the pneumococcal vaccine with the risk of developing SARS-CoV-2 infection and disease severity or risk of death on COVID-19 patients. In order to elucidate this discrepancy, we conducted a systematic review and meta-analysis to assess the association between seasonal influenza vaccination, pneumococcal vaccination and SARS-CoV-2 infection as well as with clinical outcomes related to COVID-19 morbidity and mortality. Our study followed all the available guidelines and ultimately included 38 publications that have evaluated the association of interest (24 studies on SARS-CoV-2 infection). Overall, our results indicate that influenza vaccination seems to be associated with lower risk for COVID-19 infection, as well as against hospitalization and mechanical ventilation, but no significant effect was found against “harder” endpoints like ICU admission and mortality. Specifically, the results of the meta-analysis showed that people vaccinated between autumn 2019 to autumn 2021 had up to a 20% lower risk of COVID-19 infection. Similarly, if vaccinated individuals were infected by SARS-CoV-2, the risk of hospitalization would be reduced by 12% and the need for mechanical ventilation by 27%. As far as the pneumococcal vaccination is concerned, the results show that it has a protective effect against SARS-CoV-2 infection, while it does not seem to have the same effect on COVID-19 hospitalization or ICU admission, although these analyses included a small number of studies.

During the last year, two systematic reviews and meta-analyses were published on the same topic, but the included studies were fewer than the ones included in our meta-analysis [67,68]. In the study of Wang et al. [67] a total of 9, 3, 2, and 3 studies were included for the association of influenza vaccination and SARS-CoV-2 infection, hospitalization, ICU admission and mortality, respectively. The authors stated in their results that “the association between influenza vaccination and COVID-19 clinical outcomes was not statistically significant by random effects model while the results by fixed effects model was somehow significant”. They attribute this result to “the substantial heterogeneity between the small number of studies and participants involved in each outcome”. In their meta-analysis, Zeynali Bujani et al. [68] included nine studies for the association of influenza vaccine and COVID-19, among which were two studies excluded from our meta-analysis. The particular studies of Jedi et al. [69] and Caban-Marntinez et al. [70], were excluded, as they reported effect sizes based on crude counts. Such data clearly violated our inclusion criteria, as we attempted to minimize the potential confounding by using only adjusted estimates. In addition, the study of Skowronski et al. [71] is a retrospective analysis from Canada that involved specimens collected during the 2010–2011 to 2016–2017 seasons, when specimens were tested for both influenza and non-influenza respiratory viruses (NIRVs), including some seasonal coronaviruses but not specific and exclusively SARS-CoV-2.

We performed the meta-analysis following strict criteria, and we included all the available data and investigated every study level covariate that may have influenced the outcome. Nevertheless, the between studies heterogeneity was high and the attempts to explain it using study-level covariates did not reveal anything of importance. In any case, heterogeneity is expected for many reasons that include the design of the study, the studied population, the sampling method, as well as the testing and vaccination policies in each country. In our analysis we included studies in which the seasonal influenza vaccination was administered between 2019 and 2021. The type of influenza vaccination and the exact date when the vaccines were administered was not specified in all studies, and as influenza varies across years, the results should be interpreted with caution regarding different influenza seasons. The testing policy implemented in each country also varied in the included studies, using different diagnostic tests. Most of the studies used molecular diagnosis with rt-PCR, and a few used antibody testing, whereas some studies did not specify the laboratory method used. Furthermore, we must consider that national influenza immunization policies can vary significantly from country to country. These differences arise from insufficient information on the relevance of influenza infection from a clinical, social and economic point of view [72].

Our findings suggest that both vaccines are associated with a lower risk of SARS-CoV-2 infection, but not with “harder” endpoints such as ICU admission and death. The influenza vaccine was additionally found to be associated with decreased risk for hospitalization and the need for mechanical ventilation. This association is counter-intuitive at first sight and hence needs to be explained. It is worth mentioning that the BCG vaccine, a vaccine primarily used against tuberculosis (TB),which is mandatory in several countries [33], seems also to have a protective effect since a meta-analysis of data from 160 countries has shown that when the BCG vaccine coverage was over 70% there was a reduction in the COVID-19 infections [12]. In addition, some studies have found that mandatory BCG vaccination is associated with a flattening of the curve in the spread of COVID-19 [73], and that differences in mortality produced by COVID-19 across countries are correlated with a country’s BCG vaccination policy [74,75]. However, these data come from ecological studies and thus need to be interpreted with caution.

Cross-reactivity is unlikely to exist between SARS-CoV-2 and influenza viruses, and thus other potential explanations should be considered. There is, for instance, some evidence that vaccines developed for a specific pathogen may have a wider role in protection against unrelated pathogens. Such cases include Bacillus Calmette-Guérin (BCG), measles and influenza [76]. The underlying mechanisms for such non-specific actions remain poorly understood, but a plausible suggested mechanism includes the induction of innate immune response following live attenuated vaccination in a way that is independent of memory cells (T or B cells). This type of immunity, termed “trained innate immunity”, may confer non-specific protection against different pathogens by inducing the upregulation of recognition receptors (such as toll-like receptors) and the secretion of proinflammatory cytokines (such as TNF-alpha and IL-6) in peripheral blood leucocytes and functional changes in natural killer cells [50,77].

Nevertheless, other more plausible explanations should not be ruled out. The studies included in this meta-analysis are heterogeneous in many respects including their design, the study population and the inclusion criteria. In terms of their design, all included studies were observational, so the risk of possible confounding should be considered. In such cases, when some important risk factors do exist (such as age, gender, and comorbidities), it would be unwise to perform the meta-analysis including crude estimates. However, even though we included only adjusted estimates, or studies with matched groups, the factors for which the adjustment were performed were not the same across studies. For instance, most studies adjusted for age and gender, others for several comorbidities (and even in this case, not for the same comorbidities), and some others for additional factors such as area of residence or socioeconomic status. In the subgroup analysis we tried to investigate this effect, but we were unable to find systematic differences across studies.

Socioeconomic factors, such as educational level, household size and income have been reported to have a significant effect on the probability of being vaccinated against influenza [78], whereas the same factors have been consistently shown to play a significant role in COVID-19 morbidity and mortality [79,80,81]. It is possible therefore to speculate that individuals who get vaccinated may pay more attention to their health status and lifestyle, and subsequently, they might have been more compliant to the various non-pharmaceutical intervention suggested for COVID-19 prevention, such as social distancing, wearing masks, hand hygiene practices and use of protective equipment, leading to a reduced potential risk of infection. Following the same rationale, since vaccinated individuals are under-represented in the lower socioeconomic categories (e.g., those who cannot work from home, those who need to use public transportation, or live in crowded households and so on), then these factors might explain the observed increased risk of getting COVID-19. This interpretation is also consistent with the results suggesting that vaccinated individuals are at increased risk of SARS-CoV-2 infection, but not at risk of ICU admission or death, since in these cases the comparison was made among those that were already infected, and the supposed confounding effect has been potentially removed.

Interestingly, when the analysis was restricted to the studies that adjusted for age, gender, comorbidities and some indices of socioeconomic status (such as area of residence, income or education), the association of influenza vaccination with the risk of SARS-CoV-2 infection was diminished. Thus, future and more carefully designed studies that will adjust more thoroughly for socioeconomic indices should be pursued to investigate this hypothesis. However, this observation does not seem to hold regarding the association of pneumococcal vaccination with SARS-CoV-2 infection, since the statistically significant association persists even after restricting the analysis in the studies that adjusted for the same factors. We need to mention that even though the studies regarding pneumococcal vaccination were fewer in total, the majority of them provided estimates adjusted for socioeconomic indices, which is in contrast to what is the case for studies of the influenza vaccine.

Most respiratory viral diseases may have as a complication bacterial coinfections, or secondary infections (super-infections), which can worsen the clinical outcome increasing thus morbidity and mortality. However, it has been argued that the proportion of COVID-19 patients with bacterial coinfections and/or super-infections may be lower compared to what we have seen in patients suffering from influenza [82,83,84]. Nevertheless, since vaccination is beneficial per se, it has been suggested that pneumococcal vaccination can, to some extent, provide additional protection to COVID-19 patients, reducing the morbidity and mortality [85]. Animal studies have also shown that an initial SARS-CoV-2 infection can increase susceptibility and pathogenicity to bacterial coinfection [86]. The limited number of available studies regarding the association of pneumococcal vaccination with hospitalization, ICU admission and death did not allow us to test this hypothesis, but as we said the association of vaccination with SARS-CoV-2 infection remained significant even after adjustment for gender, age, comorbidities and socioeconomic indices, and this should be investigated in future studies.

## 5. Conclusions

Overall, we conducted a systematic review and meta-analysis to assess the association between seasonal influenza vaccination and pneumococcal vaccination with SARS-CoV-2 infection and its clinical outcomes. Influenza and pneumococcal vaccination were found to be associated with a lower risk of SARS-CoV-2 infection. Regarding influenza vaccination, it seems that the majority of studies did not properly adjust for all potential confounders, so when the analysis was limited to studies that adjusted for age, gender, comorbidities and socioeconomic indices, the association diminished. This is not the case regarding the pneumococcal vaccination, for which the association persisted, even after adjustment for such factors. Regarding harder endpoints such as ICU admission and death, current data do not support the association. Possible explanations were discussed, including trained immunity, inadequate matching for socioeconomic indices and possible coinfection. Future more carefully conducted studies, at least regarding matching for socio-economic factors, should be undertaken.

## Figures and Tables

**Table 1:** Baseline characteristics of the 21 publications, involving 22 studies that assessed the association between influenza vaccination and SARS-CoV-2 infection.

First Author’s Name	Year of Publication	Country	Identification of COVID-19	Sample Size	Effect Size	Adjusted Estimate
M. Noale et al. [38]	2020	Italy	rt-PCR	6061	OR	0.85 (95% CI: 0.74–0.98)
M. Noale et al. [38]	2020	Italy	rt-PCR	619	OR	0.87 (95% CI: 0.59–1.28)
I. Martínez-Baz et al. [29]	2020	Spain	rt-PCR or Antibody Rapid test	9745	OR	1.07(95% CI: 0.92–1.24)
P. Ragni et al. [40]	2020	Italy	rt-PCR	17,608	OR	0.89 (95% CI: 0.80–0.99)
M. Belingheri et al. [31]	2020	Italy	rt-PCR	3520	OR	0.41 (95% CI: 0.07–2.39)
A. Vila-Córcoles et al. [41]	2020	Spain	rt-PCR	79,083	HR	1.02 (95% CI: 0.79–1.32)
I. Green et al. [42]	2020	Israel	rt-PCR	19,089	OR	0.79 (95% CI: 0.67–0.98)
E. Kissling et al. [15]	2021	Europe	rt-PCR	2147	OR	0.93 (95% CI: 0.66–1.32)
A. Conlon et al. [43]	2021	USA	rt-PCR	27,201	OR	0.76 (95% CI: 0.68–0.86)
B. Erismis et al. [33]	2021	Turkey	Not specified	203	RR	0.83 (95% CI: 0.75–0.93)
A. Bozek et al. [44]	2021	Poland	rt-PCR	2558	HR	0.74 (95% CI: 0.54–0.89)
M. Kowalska et al. [45]	2021	Poland	IgG antibodies	5376	OR	0.68 (95% CI: 0.55–0.83)
M. Fernández-Prada et al. [46]	2021	Spain	rt-PCR	188	OR	1.70 (95% CI: 0.97–3.25)
K. Huang et al. [37]	2021	USA	Not specified	55,667.997	OR	0.76 (95% CI: 0.75–0.77)
J. P. King et al. [47]	2021	USA	rt-PCR	1356	OR	0.83 (95% CI: 0.63–1.10)
C. Pawlowski et al. [48]	2021	USA	rt-PCR	12,791	RR	0.85 (95% CI: 0.75–0.96)
S. Caratozzolo et al. [36]	2020	Italy	rt-PCR/or infection according to WHO definition	848	OR	0.47 (95% CI: 0.29–0.74)
M. N. Rivas et al. [32]	2021	USA	IgG antibodies	6087	OR	1.84 (95% CI: 0.57–11.3)
J. G. Zein et al. [49]	2020	USA	Not specified	13,220	OR	0.79 (95% CI: 0.62–1.00)
P. A. Debisarun et al. [50]	2020	Netherlands	rt-PCR	6856	RR	0.61 (95% CI: 0.46–0.82)
Y. Xiang et al. [51]	2020	UK	Laboratory confirmed	30,835	OR	0.60 (95% CI: 0.53–0.68)
M. Alkathlan et al. [52]	2021	Saudi Arabia	Laboratory confirmed	424	OR	0.83 (95% CI: 0.51–1.35)

OR = adjusted odds ratio, HR = hazard odds ratio, RR = relative risk, rt-PCR = Reverse transcription polymerase chain reaction.

**Table 2:** Baseline characteristics of the 17 publications involving studies that assessed the association between influenza vaccination and COVID-19 clinical outcomes: (15 studies for hospitalization, four for Mechanical Ventilation/invasive respiratory support, 11 for Intensive care and 18 for mortality).

First Author’s Name	Year of Publication	Country	Identification of COVID-19	Sample Size	Effect Size	Adjusted Estimate
**Hospitalization**						
P. D. Pedote et al. [53]	2021	Italy	rt-PCR	662	OR	1.20 (95% CI: 0.70–1.90)
R. Pastorino et al. [39]	2021	Italy	rt-PCR	741	OR	1.03 (95% CI: 0.66–1.62)
C. Pawlowski et al. [48]	2021	USA	rt-PCR	959	RR	1.10 (95% CI: 0.83–1.50)
M. Gobbato et al. [54]	2020	Italy	Laboratory confirmed	3010	OR	0.78 (95% CI: 0.61–1.01)
M-J. Yang et al. [55]	2020	USA	Laboratory confirmed	2005	OR	0.41 (95% CI: 0.28–0.59)
S. Greco et al. [56]	2021	Italy	rt-PCR	952	OR	1.44 (95% CI: 1.01–2.05)
A. Bozek et al. [44]	2021	Poland	rt-PCR	151	HR	0.48 (95% CI: 0.36–0.77)
K. Huang et al. [37]	2021	USA	Not specified	55,667,977	OR	0.76 (95% CI: 0.75–0.77)
M. Massari et al. [57]	2021	Italy	rt-PCR	115,945	RR	0.87 (95% CI: 0.86–0.88)
A. Conlon et al. [43]	2021	USA	rt-PCR	1218	OR	0.58 (95% CI: 0.46–0.73)
P. Ragni et al. [40]	2020	Italy	rt-PCR	4485	HR	1.00 (95% CI: 0.84–1.19)
C. R. Wilcox et al. [58]	2021	UK	rt-PCR	6921	HR	0.85 (95% CI: 0.75–0.97)
J. G. Zein et al. [49]	2020	USA	Not specified	1434	OR	1.29 (95% CI: 0.72–2.31)
I. Ilic et al. [59]	2020	Serbia	rt-PCR	107	OR	1.31 (95% CI: 0.54–3.17)
S. M. Taghioff et al. [60]	2021	Netherlands	Not specified	74,754	OR	1.07 (95% CI: 0.96–1.18)
**Mechanical Ventilation/Invansive Respiratory Support**						
G. Fink et al. [61]	2020	Brazil	rt-PCR	39,745	OR	0.83 (95% CI: 0.77–0.89)
A. Conlon et al. [43]	2021	USA	rt-PCR	1218	OR	0.45 (95% CI: 0.27–0.78)
A. E. Demkina et al. [62]	2020	Russia	Clinically confirmed/ rt-PCR	214,751	OR	0.74 (95% CI: 0.54–1.01)
A. Bozek et al. [44]	2021	Poland	rt-PCR	21	OR	0.42 (95% CI: 0.02–9.96)
**Intensive Care**						
M-J. Yang et al. [55]	2021	USA	rt-PCR	2005	OR	0.31 (95% CI: 0.07–0.85)
M. Massari et al. [57]	2021	Italy	rt-PCR	111,740	RR	1.01 (95% CI: 0.99–1.04)
R. Pastorino et al. [39]	2021	Italy	rt-PCR	99	OR	1.26 (95% CI: 0.74–2.21)
A. Conlon et al. [43]	2021	USA	rt-PCR	1218	OR	0.64 (95% CI: 0.41–1.00)
C. Pawlowski et al. [48]	2021	USA	rt-PCR	959	RR	1.10 (95% CI: 0.56–2.20)
G. Fink et al. [61]	2020	Brazil	rt-PCR	39,156	OR	0.93 (95% CI: 0.87–0.99)
A. E. Demkina et al. [62]	2020	Russia	Clinically confirmed/ rt-PCR	214,751	OR	0.76 (95% CI: 0.59–0.97)
M. Candelli et al. [63]	2021	Italy	rt-PCR	602	OR	0.73 (95% CI: 0.35–1.56)
M. L. de la Cruz Contyet al. [34]	2021	Spain	rt-PCR	206	OR	1.92 (95% CI: 0.36–10.3)
J. G. Zein et al. [49]	2020	USA	Not specified	1434	OR	0.65 (95% CI: 0.22–1.79)
S. M. Taghioff et al. [60]	2021	Netherlands	Not specified	74,754	OR	1.18 (95% CI: 1.00–1.39)
**Mortality**						
S. Greco et al. [56]	2021	Italy	rt-PCR	952	OR	1.06 (95% CI: 0.60–1.88)
P. D. Pedote et al. [53]	2021	Italy	rt-PCR	662	OR	1.60 (95% CI: 0.80–3.20)
R. Pastorino et al. [39]	2021	Italy	rt-PCR	97	OR	1.33 (95% CI: 0.77–2.31)
M. Massari et al. [57]	2021	Italy	rt-PCR	115,945	RR	1.04 (95% CI: 1.01–1.06)
J. M. Fernadez. Ibánez et al. [64]	2021	Spain	rt-PCR	410	OR	1.55 (95% CI: 0.96–2.48)
A. Bozek et al. [44]	2021	Poland	rt-PCR	2558	HR	0.74 (95% CI: 0.03–20.8)
M. Candelli et al. [63]	2021	Italy	rt-PCR	602	OR	0.20 (95% CI: 0.08–0.51)
A. Conlon et al. [43]	2021	USA	rt-PCR	1218	HR	0.94 (95% CI: 0.61–1.47)
P. Ragni et al. [40]	2020	Italy	rt-PCR	4872	HR	1.14 (95% CI: 0.95–1.37)
C. R Wilcox et al. [58]	2021	UK	rt-PCR	6368	OR	0.76 (95% CI: 0.64–0.90)
G. Fink et al. [61]	2020	Brazil	rt-PCR	53,752	OR	0.84 (95% CI: 0.78–0.91)
A. E. Demkina et al. [62]	2020	Russia	Clinically confirmed/ rt-PCR	117,346	HR	0.78 (95% CI: 0.63–0.95)
Y. Azzi et al. [35]	2020	USA	rt-PCR	229	OR	0.88 (95% CI: 0.70–0.96)
M. Gobbato et al. [54]	2020	Italy	Laboratory confirmed	3010	OR	0.78 (95% CI: 0.61–1.01)
J. G. Zein et al. [49]	2020	USA	Not specified	14,654	OR	0.98 (95% CI: 0.39–2.43)
D. Giannoglou et al. [65]	2020	Greece	Not specified	512	OR	0.38 (95% CI: 0.17–0.81)
E. Ortiz-Prado et al. [66]	2020	Ecuador	rt-PCR	9468	RR	1.40 (95% CI: 0.46–4.28)
S. M. Taghioff et al. [57]	2021	Netherlands	Not specified	74,754	OR	0.89 (95% CI: 0.77–1.03)

**Table 3:** Baseline characteristics of (a) six publications, involving 10 studies, that assessed the association between pneumococcal vaccination and SARS-CoV-2 infection, (b) four studies that assessed the association between pneumococcal vaccination and the risk of hospitalization, and (c) three studies that assessed the association between pneumococcal vaccination and the risk of requiring intensive care.

First Author’s Name	Year of Publication	Country	Identification of COVID-19	Sample Size	Effect Size	Adjusted Estimate
**Infection**						
A. Vila-Córcoles et al. [41]	2020	Spain	rt-PCR	79,083	HR	1.02 (95% CI0.78–1.33)
M. Noale et al. [38]	2020	Italy	rt-PCR	6061	OR	0.61 (95% CI0.41–0.91)
M. Noale et al. [38]	2020	Italy	rt-PCR	619	OR	0.56 (95% CI0.33–0.95)
C. Pawlowski et al. [48]	2021	USA	rt-PCR	4693	RR	0.72 (95% CI0.56–0.92)
C. Pawlowski et al. [48]	2021	USA	rt-PCR	4636	RR	1.06 (95% CI0.81–137)
M. N. Rivas et al. [32]	2021	USA	IgG antibodies	6083	OR	0.99 (95% CI0.71–1.36)
M. Fernández-Prada et al. [46]	2021	Spain	rt-PCR	188	OR	0.40 (95% CI0.17–1.01)
M. Fernández-Prada et al. [46]	2021	Spain	rt-PCR	187	OR	0.70 (95% CI: 0.28–2.10)
M. Fernández-Prada et al. [46]	2021	Spain	rt-PCR	188	OR	0.20 (95% CI: 0.06–1.18)
Y. Xiang et al. [51]	2020	UK	Laboratory confirmed	30,835	OR	0.5 (95% CI: 0.31–0.82)
**Hospitalization**						
M. Gobbato et al. [54]	2020	Italy	Laboratory confirmed	3010	OR	1.53 (95% CI:1.91–1.97)
R. Pastorino et al. [39]	2021	Italy	rt-PCR	741	OR	0.96 (95% CI: 0.53–1.78)
C. Pawlowski et al. [48]	2021	USA	rt-PCR	277	RR	1.30 (95% CI: 0.86–2.10)
C. Pawlowski et al. [48]	2021	USA	rt-PCR	282	RR	1.20 (95% CI: 0.71–2.20)
**Intensive Care Unit**						
R. Pastorino et al. [39]	2021	Italy	rt-PCR	741	OR	0.75 (95% CI: 0.35–1.61)
C. Pawlowski et al. [48]	2021	USA	rt-PCR	254	RR	1.40 (95% CI: 0.48–3.90)
C. Pawlowski et al. [48]	2021	USA	rt-PCR	236	RR	1.10 (95% CI: 0.46–2.40)

**Table 4:** Effect sizes, heterogeneity statistics and results for publication bias on the association between influenza vaccination, SARS-CoV-2 infection as well as clinical outcomes.

Outcomes	No. of Studies	Adjusted Estimates (95% CI)	*p*-Value	*I*^2^ Value (%)	Bias (*p*-Value)
SARS-CoV-2 infection	22	0.80 (0.75–0.86)	<0.01	70.1	Begg’s test: 0.82 Egger’s test: 0.19
Hospitalization	15	0.88 (0.81–0.95)	<0.01	95.1	Begg’s test: 0.55 Egger’s test: 0.62
Mechanical Ventilation/invasive respiratory support	4	0.73 (0.58–0.92)	0.01	42.4	Begg’s test: 0.73 Egger’s test: 0.21
Intensive care unit admission	11	0.96 (0.88–1.06)	0.40	86.0	Begg’s test: 0.76 Egger’s test: 0.14
Mortality	18	0.90 (0.81–1.01)	0.07	78.2	Begg’s test: 0.82 Egger’s test: 0.07

**Table 5:** Subgroup analysis of the association between influenza vaccination and SARS-CoV-2 infection.

Subgroup Variables	No. of Studies	Adjusted Estimate (95% CI)	*p*-Value	*I^2^* Value (%)
**Study Design**				
cross sectional study	5	0.76 (0.75–0.77)	<0.01	0.00
retrospective cohort study	10	0.80 (0.75–0.86)	<0.01	41.5
prospective cohort study	4	0.80 (0.56–1.08)	0.14	90.9
case-control study	3	0.99 (0.76–1.23)	0.93	51.7
**Study Population**				
adults-general population	13	0.80 (0.74–0.87)	<0.01	68.4
older adults	3	0.72 (0.56–0.92)	0.01	53.8
health workers	6	0.84 (0.68–1.04)	0.12	66.6
**Effect Size**				
OR	17	0.80 (0.74–0.87)	<0.01	72.5
RR/HR	5	0.82 (0.73–0.92)	<0.01	46.4
**Adjustment Factors**				
age/gender/age and gender	5	0.86 (0.62–1.21)	0.40	63.5
age, gender and comorbidities	9	0.82 (0.73–0.92)	<0.01	78.4
age, gender, comorbidities, and socioeconomic status, education or residence	6	0.87 (0.68–1.11)	0.26	81.8
**COVID-19 test**				
rt-PCR	15	0.84 (0.77–0.92)	<0.01	57.7
IgG/IgM antibodies	2	0.83 (0.38–1.82)	0.64	37.9
Laboratory confirmed (not specified)	5	0.74 (0.66–0.83)	<0.01	74.9
**Continent**				
Europe	13	0.81 (0.70–0.93)	<0.01	79.7
N. America	6	0.76 (0.75–0.77)	<0.01	0.00
Asia	3	0.83 (0.77–0.90)	<0.01	0.00

**Table 6:** Subgroup analysis of the association between influenza vaccination and COVID-19 clinical outcomes.

Subgroup Variables	No. of Studies	Adjusted Estimate (95% CI)	*p*-Value	*I*^2^ Value (%)
**Hospitalization**				
**Effect Size**				
OR	10	0.92 (0.78–1.02)	0.40	86.6
RR/HR	5	0.87 (0.71–0.99)	0.03	71.0
**Adjusted Factors**				
age/gender/age and gender	1	1.44 (1.00–2.07)	0.05	89.8
age, gender and comorbidities	7	0.85 (0.71–1.03)	0.49	66.3
**COVID-19 test**				
rt-PCR	10	0.89 (0.78–1.02)	0.09	74.6
Laboratory confirmed/ (not specified)	5	0.88 (0.67–1.12)	0.30	90.0
**Continent**				
Europe	10	0.93 (0.83–1.04)	0.21	75.7
N. America	5	0.80 (0.63–1.02)	0.07	73.1
**Intensive Care Unit**				
**Effect Size**				
OR	9	0.91 (0.76–1.08)	0.26	55.9
HR	2	1.01 (0.99–1.04)	0.43	0.0
COVID-19 test				
rt-PCR	9	0.93 (0.84–1.03)	0.14	58.5
Laboratory confirmed/ (not specified)	2	1.12 (0.80–1.57)	0.52	14.1
**Adjusted Factors**				
age, gender and comorbidities	4	0.87 (0.65–1.17)	0.35	17.6
**Continent**				
Europe	5	1.05 (0.95–1.16)	0.32	20.6
N. America	4	0.69 (0.47–1.02)	0.06	10.3
S. America	1	0.93 (0.87–0.99)	0.02	-
Asia	1	0.76 (0.59–0.98)	0.03	-
**Mortality**				
**Effect Size**				
OR	12	0.87 (0.76–0.99)	0.03	60.5
HR	6	0.99 (0.86–1.12)	0.82	46.3
**COVID-19 test**				
rt-PCR	14	0.93 (0.82–1.06)	0.27	80.3
Laboratory confirmed/ (not specified)	4	0.81 (0.65–1.00)	0.05	37.1
**Adjusted Factors**				
age/gender/age and gender	3	1.07 (0.75–1.53)	0.70	59.2
age, gender and comorbidities	8	0.82 (0.61–1.10)	0.19	75.3
**Continent**				
Europe	12	0.94 (0.80–1.09)	0.40	75.3
N. America	3	0.89 (0.76–1.03)	0.12	0.0
S. America	2	0.84 (0.78–0.91)	<0.01	0.0
Asia	1	0.78 (0.63–0.95)	0.02	-

**Table 7:** Effect sizes, heterogeneity statistics and results for publication bias of the association between pneumococcal vaccination and SARS-CoV-2 infection and outcomes.

Outcomes	No. of Studies	Adjusted Estimates (95% CI)	*p*-Value	*I*^2^ value (%)	Bias (*p*-Value)
SARS-CoV-2 infection	10	0.70 (0.57–0.88)	<0.01	47.2	Begg’s test: 0.37 Egger’s test: 0.11
Hospitalization	4	1.47 (1.30–1.67)	<0.01	11.4	Begg’s test: 0.09 Egger’s test: 0.05
Intensive care unit admission	3	0.99 (0.60- 1.64)	0.96	0.0	Begg’s test: 0.30 Egger’s test: 0.30

## Data Availability

Data are available in the tables.

## Supplementary Materials

The following supporting information can be downloaded at: https://www.mdpi.com/article/10.3390/diagnostics12123086/s1, Table S1. Baseline characteristics of the 21 publications, involving 22 studies that assessed the association between influenza vaccination and SARS-CoV-2 infection [15,29,31,32,33,36,37,38,40,42,43,44,45,46,47,48,49,50,51,52,60]. Table S2. Baseline characteristics of the 17 publications involving studies that assessed the association between influenza vaccination and COVID-19 clinical outcomes: (15 studies for hospitalization, 4 for Mechanical Ventilation/ invasive respiratory support, 11 for Intensive care and 18 for mortality) [34,35,37,39,40,43,44,48,49,53,54,55,56,57,58,59,60,61,62,63,64,65,66]. Table S3. Baseline characteristics of (a) 6 publications, involving 10 studies, that assessed the association between pneumococcal vaccination and SARS-CoV-2 infection, (b) 4 studies that assessed the association between pneumococcal vaccination and the risk of hospitalization and (c) 3 studies that assessed the association between pneumococcal vaccination and the risk of intensive care [32,38,39,41,46,48,51,54]. Table S4. Baseline characteristics of the studies excluded from the meta-analysis that assessed the association between influenza vaccination and SARS-CoV-2 infection or its outcome [30,36,69,70,87,88,89,90,91,92,93,94,95,96,97,98,99,100].
