# Supplementary material for: Influenza and Pneumococcal Vaccination and the Risk of COVID-19: A Systematic Review and Meta-Analysis
Source: Diagnostics (Basel). 2022 Dec 7;12(12):3086. doi: 10.3390/diagnostics12123086 (PMC9776999; doi:10.3390/diagnostics12123086)
Supplement: Supplementary file 1 [file diagnostics-12-03086-s001.zip › diagnostics-1993617-supplementary.pdf]

Table S1. *Baseline characteristics of the 21 publications, involving 22 studies that assessed the association between influenza vaccination and SARS-CoV-2 infection.*

First Author's name	Year of publication	Country	Study design/ Study population	Identification of COVID-19	Sample size	Effect size	Adjusted estimate	Adjusted factors
M. Noale et al. [1]	2020	Italy	Cross-sectional study/ Adults-general population	rt-PCR	6,061	OR	0.85 (95% CI: 0.74-0.98)	age, gender, self-reported comorbidities,education, area of residence, smoking status
M. Noale et al. [1]	2020	Italy	Cross-sectional study/ Old adults	rt-PCR	619	OR	0.87 (95% CI: 0.59-1.28)	gender, age, self-reported comorbidities, education, area of residence, smoking status
I. Martínez-Baz et al. [2]	2020	Spain	Prospective cohort study/ Health workers	rt-PCR or Antibody Rapid test	9,745	OR	1.07(95% CI: 0.92-1.24)	age, gender, major chronic conditions, profession, and any ILI diagnosis in the previous five years.
P. Ragni et al. [3]	2020	Italy	Case control study/ Adults-general population	rt-PCR	17,608	OR	0.89 (95% CI: 0.80-0.99)	age, gender, Charlson index score and time of the swab test
M. Belingheri et al. [4]	2020	Italy	Retrospective cohort study/ Health workers	rt-PCR	3,520	OR	0.41 (95% CI: 0.07-2.39)	age, gender, number of flu vaccination uptakes
A. Vila-Córcoles et al. [5]	2020	Spain	Retrospective cohort study/ Adults-general population	rt-PCR	79,083	HR	1.02 (95% CI: 0.79-1.32)	age, gender, comorbidities, residence and vaccination history
I. Green et al. [6]	2020	Israel	Retrospective cohort study/ Adults-general population	rt-PCR	19,089	OR	0.79 (95% CI: 0.67-0.98)	age, gender, ethnicity, underlying medical conditions, obesity, and smoking status
E. Kissling et al. [7]	2021	Europe	Case-control study (multicenter)/ Adults-general population	rt-PCR	2,147	OR	0.93 (95% CI: 0.66-1.32)	age, gender and chronic conditions, time of swab test
A. Conlon et al. [8]	2021	USA	Retrospective cohort study/ Adults-general population	rt-PCR	27,201	OR	0.76 (95% CI: 0.68-0.86)	age, gender, ethnicity, race, BMI, Elixhauser score, comorbidities, smoking status

B. Erismis et al. [9]	2021	Turkey	Retrospective cohort study/ Health workers	Not specified	203	RR	0.83 (95% CI: 0.75-0.93)	gender
A. Bozek et al. [10]	2021	Poland	Retrospective cohort study (multicenter)/ Adults-general population	rt-PCR	2,558	HR	0.74 (95% CI: 0.54-0.89)	age ,gender, comorbidities, high economic status,BCG vaccination in the past
M. Kowalska et al. [11]	2021	Poland	Cross-sectional study/ Adults-general population	IgG antibodies	5,376	OR	0.68 (95% CI: 0.55-0.83)	age, gender, comorbidities, previous COVID-19 contact or quarantine
M. Fernández-Prada et al. [12]	2021	Spain	Case-control study/ Adults-general population	rt-PCR	188	OR	1.70 (95% CI: 0.97-3.25)	age, gender, patient location
K. Huang et al. [13]	2021	USA	Cross-sectional study/ Old adults	Not specified	55,667,997	OR	0.76 (95% CI: 0.75-0.77)	age, gender, comorbidities
J. P. King et al. [14]	2021	USA	Prospective cohort study/ Adults-general population	rt-PCR	1,356	OR	0.83 (95% CI: 0.63-1.10)	age,sample collection interval, gender, high-risk condition and month of onset
C. Pawlowski et al. [15]	2021	USA	Retrospective cohort study/ Adults-general population	rt-PCR	12,791	RR	0.85 (95% CI: 0.75-0.96)	age, gender, race, ethnicity, county level COVID-19 incidence rate, county-level COVID-19 test positive rate, Elixhauser, comorbidities, pregnancy, number of other vaccines
S. Caratozzolo et al. [16]	2020	Italy	Retrospectivecohortstudy/ Old adults	rt-PCR/ or infection according to WHO definition	848	OR	0.47 (95% CI: 0.29-0.74)	age, gender, comorbidities, clinical dementia rating scale
M. N. Rivas et al. [17]	2021	USA	Retrospectivecohort study/ Health workers	IgG antibodies	6,087	OR	1.84 (95% CI: 0.57-11.3)	age, gender
J. G. Zein et al. [18]	2020	USA	Prospective cohort study/	Not specified	13,220	OR	0.79 (95% CI: 0.62-1.00)	age, gender, race and comorbidities

			Adults-general population					
P. A. Debisarun et al. [19]	2020	Netherlands	Retrospective cohort study/ Health workers	rt-PCR	6,856	RR	0.61 (95% CI: 0.46-0.82)	age
Y. Xiang et al. [20]	2020	UK	Prospective cohort study/ Adults-general population	Laboratoryconfirmed	30,835	OR	0.60 (95% CI: 0.53-0.68)	age, gender, ethnic group,comorbidities,socioeconomic status, smoking status, anthropometric measures
M.Alkathlan et al. [21]	2021	Saudi Arabia	Cross-sectional study/ Health workers	Laboratory confirmed	424	OR	0.83 (95% CI: 0.51-1.35)	age, gender, comorbidities, nationality, educational level, flu vaccine efficacy

OR=adjusted odds ratio, HR=hazard odds ratio, RR=relative risk, rt-PCR=Reverse transcription polymerase chain reaction

Table S2. Baseline characteristics of the 17 publications involving studies that assessed the association between influenza vaccination and COVID-19 clinical outcomes: (15 studies for hospitalization, 4 for Mechanical Ventilation/ invasive respiratory support, 11 for Intensive care and 18 for mortality).

First Author's name	Year of publication	Country	Study design	Identification of COVID-19	Sample size	Effect size	Adjusted estimate	Adjusted factors
**Hospitalization**								
P. D. Pedote et al. [22]	2021	Italy	Retrospective cohort study	rt-PCR	662	OR	1.20 (95% CI: 0.70-1.90)	age, gender, chronic disease
R. Pastorino et al. [23]	2021	Italy	Prospective cohortstudy	rt-PCR	741	OR	1.03 (95% CI: 0.66-1.62)	age, gender, comorbidities
C. Pawlowski et al. [15]	2021	USA	Retrospective cohort study	rt-PCR	959	RR	1.10 (95% CI: 0.83-1.50)	age, gender, race, ethnicity, county level COVID-19 incidence rate, county-level COVID-19 test positive rate, Elixhauser comorbidities, pregnancy, number of other vaccines
M. Gobbato et al. [24]	2020	Italy	Retrospective cohort study	Laboratory confirmed	3,010	OR	0.78 (95% CI: 0.61-1.01)	age, gender, comorbidities, medication, health district
M-J. Yang et al. [25]	2020	USA	Retrospective cohort study (single-centered)	Laboratory confirmed	2,005	OR	0.41 (95% CI: 0.28-0.59)	age, gender, ethnicity/race, comorbidities

S. Greco et al. [26]	2021	Italy	Retrospective cohort study (multicenter)	rt-PCR	952	OR	1.44 (95% CI: 1.01-2.05)	gender
A. Bozek et al. [10]	2021	Poland	Retrospective cohort study (multicenter)	rt-PCR	151	HR	0.48 (95% CI: 0.36-0.77)	age,gender,comorbidities, high economic status, BCG vaccination in the past
K. Huang et al. [13]	2021	USA	Cross-sectional study	Not specified	55,667,977	OR	0.76 (95% CI: 0.75-0.77)	age, gender, comorbidities
M. Massari et al. [27]	2021	Italy	Retrospective cohort study	rt-PCR	115,945	RR	0.87 (95% CI: 0.86-0.88)	age, gender, Charlson comorbidity index, ethnicity, drugs, comorbidities
A. Conlon et al. [8]	2021	USA	Retrospective cohort study	rt-PCR	1,218	OR	0.58 (95% CI: 0.46-0.73)	age, gender, ethnicity, race, BMI, Elixhauser score, comorbidities, smoking status
P. Ragni et al. [3]	2020	Italy	Prospective cohort study	rt-PCR	4,485	HR	1.00 (95% CI: 0.84-1.19)	age, comorbidities,exposure to influenza vaccination
C. R. Wilcox et al. [28]	2021	UK	Retrospective cohort study	rt-PCR	6,921	HR	0.85 (95% CI: 0.75-0.97)	age, gender, BMI, socioeconomic status (IMD), smoking status, frailty score (electronic frailty index), pre-existing comorbidities and medication
J. G. Zein et al. [18]	2020	USA	Prospective cohort study	Not specified	1,434	OR	1.29 (95% CI: 0.72-2.31)	age, gender, race and comorbidities
I. Ilic et al. [29]	2020	Serbia	Retrospective cohort study	rt-PCR	107	OR	1.31 (95% CI: 0.54-3.17)	age, malignancy, hypertension, coronary artery disease, heart failure, diabetes mellitus, bronchial asthma, previous CVD, arrhythmia, BMI, current smoking
S. M. Taghioff et al. [5]	2021	Netherlands	Retrospective cohort study	Not specified	74,754	OR	1.07 (95% CI: 0.96-1.18)	age, race, ethnicity, gender, hypertension, diabetes,hyperlipidemia, chronic obstructive

								pulmonary disease (COPD), obesity, heart disease, andlifestyle habits such as smoking
**Mechanical Ventilation/ Invansive Respiratory Support**								
G. Fink et al. [30]	2020	Brazil	Retrospective cohort study	rt-PCR	39,745	OR	0.83 (95% CI: 0.77-0.89)	age, gender, race, educational attainment,comorbidities, treatment facility
A.Conlon et al. [8]	2021	USA	Retrospective cohort study	rt-PCR	1,218	OR	0.45 (95% CI: 0.27-0.78)	age, gender, ethnicity, race, BMI, Elixhauser score, comorbidities, smoking status
A.E. Demkina et al. [31]	2020	Russia	Cohort study	Clinically confirmed/ rt-PCR	214,751	OR	0.74 (95% CI: 0.54-1.01)	age, patient's gender, comorbidities
A.Bozek et al. [10]	2021	Poland	Retrospective cohort study (multicenter)	rt-PCR	21	OR	0.42 (95% CI: 0.02-9.96)	age, gender, comorbidities, high economic status, BCG vaccination in the past
**Intensive Care**								
M-J. Yang et al. [25]	2021	USA	Retrospective cohort study (single-centered)	rt-PCR	2,005	OR	0.31 (95% CI: 0.07-0.85)	age, gender, ethnicity/race, comorbidities
M. Massari et al. [27]	2021	Italy	Retrospective cohort study	rt-PCR	111,740	RR	1.01 (95% CI: 0.99-1.04)	age, gender, comorbidities, ethnicity, drugs
R. Pastorino et al. [23]	2021	Italy	Prospective cohort study	rt-PCR	99	OR	1.26 (95% CI: 0.74-2.21)	age, gender, comorbidities
A. Conlon et al. [8]	2021	USA	Retrospective cohort study	rt-PCR	1,218	OR	0.64 (95% CI: 0.41-1.00)	age, gender, ethnicity, race, BMI, Elixhauser score, comorbidities, smoking status
C. Pawlowski et al. [15]	2021	USA	Retrospective cohort study	rt-PCR	959	RR	1.10 (95% CI: 0.56-2.20)	age, gender, race, ethnicity, county level COVID-19 incidence rate, county-level COVID-19 test positive rate, Elixhauser

								comorbidities, pregnancy, number of other vaccines
G. Fink et al. [30]	2020	Brazil	Retrospective cohort study	rt-PCR	39,156	OR	0.93 (95% CI: 0.87-0.99)	age, gender, race, educational attainment,comorbidities, treatment facility
A. E. Demkina et al. [31]	2020	Russia	Retrospective cohort study	Clinically confirmed/ rt-PCR	214,751	OR	0.76 (95% CI: 0.59-0.97)	age, patient'sgender, comorbidities
M. Candelli et al. [32]	2021	Italy	Retrospective cohort study	rt-PCR	602	OR	0.73 (95% CI: 0.35-1.56)	age, gender, comorbidities
M. L. de la Cruz Conty et al. [33]	2021	Spain	Prospective cohort study (multicenter)	rt-PCR	206	OR	1.92 (95% CI: 0.36-10.3)	age, gender, comorbidities
J. G. Zein et al. [18]	2020	USA	Prospective cohort study	Not specified	1,434	OR	0.65 (95% CI: 0.22-1.79)	age, gender, race and comorbidities
S. M. Taghioff et al. [5]	2021	Netherlands	Retrospective cohort study	Not specified	74,754	OR	1.18 (95% CI: 1.00-1.39)	age,race,ethnicity, gender, hypertension, diabetes,hyperlipidemia, chronic obstructive pulmonary disease (COPD), obesity, heart disease, andlifestyle habits such as smoking
**Mortality**								
S. Greco et al. [26]	2021	Italy	Retrospective cohort study	rt-PCR	952	OR	1.06 (95% CI: 0.60-1.88)	gender
P. D. Pedote et al. [22]	2021	Italy	Retrospective cohort study	rt-PCR	662	OR	1.60 (95% CI: 0.80-3.20)	age, gender, chronic diseases
R. Pastorino et al. [23]	2021	Italy	Prospective cohortstudy	rt-PCR	97	OR	1.33 (95% CI: 0.77-2.31)	age, gender, comorbidities
M.Massari et al. [27]	2021	Italy	Retrospective cohort study	rt-PCR	115,945	RR	1.04 (95% CI: 1.01-1.06)	age, gender, comorbidities, ethnicity, drugs
J. M. Fernadez. Ibánezet al. [34]	2021	Spain	Retrospective cohort study	rt-PCR	410	OR	1.55 (95% CI: 0.96-2.48)	gender, chronic diseases
A. Bozek et al. [10]	2021	Poland	Retrospective cohort study (multicenter)	rt-PCR	2,558	HR	0.74 (95% CI: 0.03-20.8)	age, gender,comorbidities, high economic status, BCG vaccination in the past
M. Candelli et al. [32]	2021	Italy	Retrospective cohort study	rt-PCR	602	OR	0.20 (95% CI: 0.08-0.51)	age, gender, comorbidities

A. Conlon et al. [8]	2021	USA	Retrospective cohort study	rt-PCR	1,218	HR	0.94 (95% CI: 0.61-1.47)	age, gender, ethnicity, race, BMI, Elixhauser score, comorbidities, smoking status
P. Ragni et al. [3]	2020	Italy	Prospective cohort study	rt-PCR	4,872	HR	1.14 (95% CI: 0.95-1.37)	age, comorbidities, exposure to influenza vaccination test
C. R Wilcox et al. [28]	2021	UK	Retrospective cohort study	rt-PCR	6,368	OR	0.76 (95% CI: 0.64-0.90)	age, gender, BMI, socioeconomic status (IMD), smoking status, frailty score (electronic frailty index), pre-existing comorbidities and medication
G. Fink et al. [30]	2020	Brazil	Retrospective cohort study	rt-PCR	53,752	OR	0.84 (95% CI: 0.78-0.91)	age, gender, race, educational attainment, comorbidities, treatment facility
A. E. Demkina et al. [31]	2020	Russia	Retrospective cohort study	Clinically confirmed/ rt-PCR	117,346	HR	0.78 (95% CI: 0.63-0.95)	age, patient'sgender, comorbidities
Y. Azzi et al. [35]	2020	USA	Prospective cohort study	rt-PCR	229	OR	0.88 (95% CI: 0.70-0.96)	age, type of kidney transplant
M. Gobbato et al. [24]	2020	Italy	Retrospective cohort study	Laboratory confirmed	3,010	OR	0.78 (95% CI: 0.61-1.01)	age, gender, comorbidities, medication, health district
J. G. Zein et al. [18]	2020	USA	Prospective cohort study	Not specified	14,654	OR	0.98 (95% CI: 0.39-2.43)	age, gender, race and comorbidities
D. Giannoglou et al. [36]	2020	Greece	Cross-sectional study	Not specified	512	OR	0.38 (95% CI: 0.17-0.81)	age, gender, comorbidities
E. Ortiz-Prado et al. [37]	2020	Ecuador	Cross-sectional study	rt-PCR	9,468	RR	1.40 (95% CI: 0.46-4.28)	age, gender, comorbidities
S. M. Taghioff et al [5]	2021	Netherlands	Retrospective cohort study	Not specified	74,754	OR	0.89 (95% CI: 0.77-1.03)	age, race, ethnicity, gender, hypertension, diabetes, hyperlipidemia, chronic obstructive pulmonary disease (COPD), obesity, heart disease, and lifestyle habits such as smoking.

Table S3. Baseline characteristics of a) 6 publications, involving 10 studies, that assessed the association between pneumococcal vaccination and SARS-CoV-2 infection, b) 4 studies that assessed the association between pneumococcal vaccination and the risk of hospitalization and c) 3 studies that assessed the association between pneumococcal vaccination and the risk of intensive care.

First Author's name	Year of publicati on	Country	Study design	Identification of COVID-19	Sample size	Effect size	Adjusted estimate	Adjusted factors
**Infection**								
A. Vila-Córcoles et al. [38]	2020	Spain	Retrospective cohort study	rt-PCR	79,083	HR	1.02 (95% CI: 0.78- 1.33)	age, gender,c omorbidities, residence and vaccination history
M. Noale et al. [1]	2020	Italy	Cross-sectional study	rt-PCR	6,061	OR	0.61 (95% CI: 0.41-0.91)	gender, age, comorbidities, education, area of residence, smoking status
M. Noale et al. [1]	2020	Italy	Cross-sectionalstudy (>65years)	rt-PCR	619	OR	0.56 (95% CI: 0.33-0.95)	gender, age, comorbidities,education, area of residence, smoking status
C. Pawlowski et al. [15]	2021	USA	Retrospective cohort study	rt-PCR	4,693	RR	0.72 (95% CI: 0.56-0.92)	age, gender, race, ethnicity, county level COVID-19 incidence rate, county-level COVID-19 test positive rate, Elixhauser comorbidities, pregnancy, number of other vaccines
C. Pawlowski et al. [15]	2021	USA	Retrospective cohort study	rt-PCR	4,636	RR	1.06 (95% CI: 0.81-137)	age, gender, race, ethnicity, county level COVID-19 incidence rate, county-level COVID-19 test positive rate, Elixhauser comorbidities, pregnancy, number of other vaccines
M. N. Rivas et al. [17]	2021	USA	Retrospective cohort study	IgG antibodies	6,083	OR	0.99 (95% CI: 0.71-1.36)	age, gender
M. Fernández-Prada et al. [12].	2021	Spain	Case-control study	rt-PCR	188	OR	0.40 (95% CI: 0.17-1.01)	age, gender and patient location

M. Fernández-Prada et al. [12]	2021	Spain	Test-negative case-control study	rt-PCR	187	OR	0.70 (95% CI: 0.28-2.10)	age, gender and patient location
M. Fernández-Prada et al. [12]	2021	Spain	Case-control study	rt-PCR	188	OR	0.20 (95% CI: 0.06-1.18)	age, gender and patient location
Y. Xiang et al. [20]	2020	UK	Prospective cohort study	Laboratory confirmed	30,835	OR	0.5 (95% CI: 0.31-0.82)	age, gender, ethnic group, comorbidities, socioeconomic status, smoking status, anthropometric measures
**Hospitalization**								
M. Gobbato et al. [24].	2020	Italy	Retrospective cohort study	Laboratory confirmed	3,010	OR	1.53 (95% CI: 1.91-1.97)	age, gender, comorbidities, medication, health district
R. Pastorino et al. [23]	2021	Italy	Prospective cohort study	rt-PCR	741	OR	0.96 (95% CI: 0.53-1.78)	age, gender, comorbidities
C. Pawlowski et al. [15]	2021	USA	Retrospective cohort study	rt-PCR	277	RR	1.30 (95% CI: 0.86-2.10)	age, gender, race, ethnicity, county level COVID-19 incidence rate, county-level COVID-19 test positive rate, Elixhauser comorbidities, pregnancy, number of other vaccines
C. Pawlowski et al. [15]	2021	USA	Retrospective cohort study	rt-PCR	282	RR	1.20 (95% CI: 0.71-2.20)	age, gender, race, ethnicity, county level COVID-19 incidence rate, county-level COVID-19 test positive rate, Elixhauser comorbidities, pregnancy, number of other vaccines
**Intensive Care Unit**								
R. Pastorino et al. [23]	2021	Italy	Prospective cohort study	rt-PCR	741	OR	0.75 (95% CI: 0.35-1.61)	age, gender, comorbidities
C. Pawlowski et al. [15]	2021	USA	Retrospective cohort study	rt-PCR	254	RR	1.40 (95% CI: 0.48-3.90)	vaccinated and unvaccinated propensity score matching; demographics (age, gender, race, ethnicity),

								county level COVID-19 incidence rate, county-level COVID-19 test positive rate, Elixhauser comorbidities, pregnancy, number of other vaccines
C. Pawlowski et al. [15]	2021	USA	Retrospective cohort study	rt-PCR	236	RR	1.10 (95% CI: 0.46-2.40)	age, gender, race, ethnicity, county level COVID-19 incidence rate, county-level COVID-19 test positive rate, Elixhauser comorbidities, pregnancy, number of other vaccines

Table S4*. Baseline characteristics of the studies excluded from the meta-analysis that assessed the association between influenza vaccination and SARS-Cov-2 infection or its outcome.*

First Author's name	Year of publication	Country	Study design	Identification of Covid-19	Sample size	Effect size	Crude estimate	Outcome	Reason of exclusion
L. Jehi et al. [39]	2020	USA	Prospetive cohort study	rt-PCR	11672 (D cohort)	Calculated OR	0.73 (95% CI: 0.63-0.84)	Infection	Crude estimate
L. Jehi et al. [39]	2020	USA	Prospetive cohort study	rt-PCR	2295 (V cohort)	Calculated OR	0.59 (95% CI: 0.40-0.88)	Infection	Crude estimate
A. J. Caban-Martinez et al. [40]	2020	USA	Cross-sectionalstudy	Rapid immunoglobulin IgM-IgG combined point-of-care (POC) lateral flow immunoassay	185	Calculated OR	0.01 (95% CI: 0.01-1.71)	Infection	Crude estimate
M. Bersanelli et al. [41]	2020	Italy	Prospective cohort study	rt-PCR	955	Calculated OR	0.73 (95% CI: 0.25-2.13)	Infection	Crude estimate
L. Mara da Silva Oliveira et al. [42]	2020	Brazil	Prospective cohort study	IgG antibodies	435	OR	0.51(95% CI: 0.29-0.87)	Infection	Crude estimate
D. Kindgen-Milles et al. [43]	2021	Germany	Prospective cohort study	Antibodies IgG/ IgM and rt-PCR	500	Calculated OR	0.97 (95% CI: 0.36-2.61)	Infection	Crude estimate
N. Massoudi et al. [44]	2021	Iran	Case-control study	rt-PCR	261	OR	0.01 (95% CI: 0.00-0.15)	Infection	
L. Jehi et al. [39]	2020	USA	Retrospective cohort study	rt-PCR	2852	Calculated OR	2.04 (95% CI: 1.70-2.45)	Hospitaliz ation	Crude estimate
L. Jehi et al. [39]	2020	USA	Retrospectivecohort	rt-PCR	1684	Calculated OR	2.46 (95% CI: 1.94-3.10)	Hospitaliz ation	Crude estimate
E. Murillo-Zamora et al. [45]	2020	Mexico	Retrospective cohort study	rt-PCR	740	N/ A	-	Hospitaliz	Data not

								ation	available
U. A. Angulo-Zamudio et al. [46]	2021	Mexico	Retrospective cohort study	rt-PCR	1737	Calculated OR	0.44 (95% CI:2.78-0.68)	Mortality	Crude estimate
S. Caratozzolo et al. [16]	2020	Italy	Retrospective cohort study	rt-PCR/ or WHO definition		N/ A	-	Mortality	Data not available
N. M. Alamdari et al. [47]	2020	Iran	Retrospective cross-sectional large-scale	rt-PCR	459	Calculated OR	0.55 (95% CI: 0.23-1.30)	Mortality	Crude estimate
C. Sanchez-Garcia et al. [48]	2021	Mexico	Retrospective cohort study	Not specified	16879	OR	0.31 (95% CI: 0.15-0.64)	Mechanical ventilation	Crude estimate
					16879	OR	0.44 (95% CI: 0.35-0.55)	Mortality	
B. Poblador-Plou et al. [49]	2020	Spain	Retrospective cohort study	Laboratory confirmed	1821	Calculated OR	3.15 (95% CI: 2.50-3.96)	Mortality	Crude etsimate
					2593		4.63 (95% CI: 3.65-5.88		
D. M. Sardinha et al. [50]	2020	Brazil	Cross-sectional study	Not specified	472688	-	0.80 (95% CI: 0.79-0.81)	Mortality	Crude estimate
					472688	-	0.75 (95% CI: 0.74-0.88)	Intensive care	
					472688	-	0.69 (95% CI: 0.67-0.71)	Mechanical ventilation / Invansiver espiratory support	
D. Marín-Hernánde et al. [51]	2021	USA	Ecological study	-	-	r	-0.59	Mortality	
						R^2	0.35		
C. Zanettini et al [52]	2020	USA	Ecological study	-		r	-3.29	Mortality	
P. Cocco et al. [53]	2021	Italy	Ecological study	-		r	0.55	Infection	
						r	0.55	Mortality	
M. C. Arokiaraj et al [54]	2020	India	Ecological study	-	-	r	-0.53	Infection	
						R^2	0.28		
						r	-0.37	Mortality	
						R^2	0.13		
M. Amato et al. [55]	2020	Italy	Ecological study	-	-	Beta	-130 (95% CI:-198;-62)	Infection	
							-4.16 (95% CI:6.27;-2.05)	Hospitalization	
							-0.58 (95% CI:1.05; -0.12)	Intensive unit	
							-3.29 (95% CI:-5.66;-0.93)	Mortality	

References

1. Noale, M., et al., *The Association between Influenza and Pneumococcal Vaccinations and SARS-Cov-2 Infection: Data from the EPICOVID19 Web-Based Survey.* Vaccines (Basel), 2020. **8**(3).
2. Martinez-Baz, I., et al., *Influenza Vaccination and Risk of SARS-CoV-2 Infection in a Cohort of Health Workers.* Vaccines (Basel), 2020. **8**(4).
3. Ragni, P., et al., *Association between Exposure to Influenza Vaccination and COVID-19 Diagnosis and Outcomes.* Vaccines (Basel), 2020. **8**(4).
4. Belingheri, M., et al., *Association between seasonal flu vaccination and COVID-19 among healthcare workers.* Occup Med (Lond), 2020. **70**(9): p. 665-671.
5. Taghioff, S.M., et al., *Examining the potential benefits of the influenza vaccine against SARS-CoV-2: A retrospective cohort analysis of 74,754 patients.* PLoS One, 2021. **16**(8): p. e0255541.
6. Green, I., et al., *The association of previous influenza vaccination and coronavirus disease-2019.* Hum Vaccin Immunother, 2021. **17**(7): p. 2169-2175.
7. Kissling, E., et al., *Absence of association between 2019-20 influenza vaccination and COVID-19: Results of the European I-MOVE-COVID-19 primary care project, March-August 2020.* Influenza Other Respir Viruses, 2021. **15**(4): p. 429-438.
8. Conlon, A., et al., *Impact of the influenza vaccine on COVID-19 infection rates and severity.* Am J Infect Control, 2021. **49**(6): p. 694-700.
9. Erismis, B., et al., *Annual influenza vaccination effect on the susceptibility to COVID-19 infection.* Cent Eur J Public Health, 2021. **29**(1): p. 14-17.
10. Bozek, A., et al., *Impact of influenza vaccination on the risk of SARS-CoV-2 infection in a middle-aged group of people.* Hum Vaccin Immunother, 2021. **17**(9): p. 3126-3130.
11. Kowalska, M., et al., *Association between Influenza Vaccination and Positive SARS-CoV-2 IgG and IgM Tests in the General Population of Katowice Region, Poland.* Vaccines (Basel), 2021. **9**(5).
12. Fernandez-Prada, M., et al., *Personal and vaccination history as factors associated with SARS-CoV-2 infection.* Med Clin (Engl Ed), 2021. **157**(5): p. 226-233.
13. Huang, K., et al., *Influenza vaccination and the risk of COVID-19 infection and severe illness in older adults in the United States.* Sci Rep, 2021. **11**(1): p. 11025.
14. King, J.P., H.Q. McLean, and E.A. Belongia, *Risk of symptomatic severe acute respiratory syndrome coronavirus 2 infection not associated with influenza vaccination in the 2019-2020 season.* Influenza Other Respir Viruses, 2021. **15**(6): p. 697-700.
15. Pawlowski, C., et al., *Exploratory analysis of immunization records highlights decreased SARS-CoV-2 rates in individuals with recent non-COVID-19 vaccinations.* Sci Rep, 2021. **11**(1): p. 4741.
16. Caratozzolo, S., et al., *The impact of COVID-19 on health status of home-dwelling elderly patients with dementia in East Lombardy, Italy: results from COVIDEM network.* Aging Clin Exp Res, 2020. **32**(10): p. 2133-2140.
17. Rivas, M.N., et al., *BCG vaccination history associates with decreased SARS-CoV-2 seroprevalence across a diverse cohort of health care workers.* J Clin Invest, 2021. **131**(2).
18. Zein, J.G., G. Whelan, and S.C. Erzurum, *Safety of influenza vaccine during COVID-19.* Journal of Clinical and Translational Science, 2021. **5**(1).
19. Debisarun, P.A., et al., *The effect of influenza vaccination on trained immunity: impact on COVID-19.* MedRxiv, 2020.
20. Xiang, Y., K.C. Wong, and H.C. So, *Exploring Drugs and Vaccines Associated with Altered Risks and Severity of COVID-19: A UK Biobank Cohort Study of All ATC Level-4 Drug Categories Reveals Repositioning Opportunities.* Pharmaceutics, 2021. **13**(9).
21. Alkathlan, M., et al., *Trends, Uptake, and Predictors of Influenza Vaccination Among Healthcare Practitioners During the COVID-19 Pandemic Flu Season (2020) and the Following Season (2021) in Saudi Arabia.* J Multidiscip Healthc, 2021. **14**: p. 2527-2536.
22. Pedote, P.D., et al., *Influenza Vaccination and Health Outcomes in COVID-19 Patients: A Retrospective Cohort Study.* Vaccines (Basel), 2021. **9**(4).
23. Pastorino, R., et al., *Influenza and pneumococcal vaccinations are not associated to COVID-19 outcomes among patients admitted to a university hospital.* Vaccine, 2021. **39**(26): p. 3493-3497.
24. Gobbato, M., et al., *Clinical, demographical characteristics and hospitalisation of 3,010 patients with Covid-19 in Friuli Venezia Giulia Region (Northern Italy). A multivariate, population-based, statistical analysis.* Epidemiol Prev, 2020. **44**(5-6 Suppl 2): p. 226-234.
25. Yang, M.J., et al., *Influenza Vaccination and Hospitalizations Among COVID-19 Infected Adults.* J Am Board Fam Med, 2021. **34**(Suppl): p. S179-S182.

26. Greco, S., et al., *SARS-CoV-2 infection and H1N1 vaccination: does a relationship between the two factors really exist? A retrospective analysis of a territorial cohort in Ferrara, Italy.* Eur Rev Med Pharmacol Sci, 2021. **25**(6): p. 2795-2801.
27. Massari, M., et al., *Association of Influenza Vaccination and Prognosis in Patients Testing Positive to SARS-CoV-2 Swab Test: A Large-Scale Italian Multi-Database Cohort Study.* Vaccines (Basel), 2021. **9**(7).
28. Wilcox, C.R., N. Islam, and H. Dambha-Miller, *Association between influenza vaccination and hospitalisation or all-cause mortality in people with COVID-19: a retrospective cohort study.* BMJ Open Respir Res, 2021. **8**(1).
29. Ilic, I., et al., *Pneumonia in medical professionals during COVID-19 outbreak in cardiovascular hospital.* Int J Infect Dis, 2021. **103**: p. 188-193.
30. Fink, G., et al., *Inactivated trivalent influenza vaccination is associated with lower mortality among patients with COVID-19 in Brazil.* BMJ Evid Based Med, 2020.
31. Demkina, A.E., et al., *Risk factors for outcomes of COVID-19 patients: an observational study of 795 572 patients in Russia.* medRxiv, 2020.
32. Candelli, M., et al., *Effect of influenza vaccine on COVID-19 mortality: A retrospective study.* Internal and emergency medicine, 2021. **16**(7): p. 1849-1855.
33. de la Cruz Conty, M.L., et al., *Impact of Recommended Maternal Vaccination Programs on the Clinical Presentation of SARS-CoV-2 Infection: A Prospective Observational Study.* Vaccines (Basel), 2021. **9**(1).
34. Fernandez Ibanez, J.M., et al., *Influence of influenza vaccine and comorbidity on the evolution of hospitalized COVID-19 patients.* Med Clin (Barc), 2021.
35. Azzi, Y., et al., *COVID-19 infection in kidney transplant recipients at the epicenter of pandemics.* Kidney Int, 2020. **98**(6): p. 1559-1567.
36. Giannoglou, D., et al., *Predictors of mortality in hospitalized COVID-19 patients in Athens, Greece.* medRxiv, 2020.
37. Ortiz-Prado, E., et al., *Epidemiological, socio-demographic and clinical features of the early phase of the COVID-19 epidemic in Ecuador.* PLoS Negl Trop Dis, 2021. **15**(1): p. e0008958.
38. Vila-Corcoles, A., et al., *Influence of prior comorbidities and chronic medications use on the risk of COVID-19 in adults: a population-based cohort study in Tarragona, Spain.* BMJ Open, 2020. **10**(12): p. e041577.
39. Jehi, L., et al., *Individualizing Risk Prediction for Positive Coronavirus Disease 2019 Testing: Results From 11,672 Patients.* Chest, 2020. **158**(4): p. 1364-1375.
40. Caban-Martinez, A.J., et al., *Epidemiology of SARS-CoV-2 antibodies among firefighters/paramedics of a US fire department: a cross-sectional study.* Occup Environ Med, 2020. **77**(12): p. 857-861.
41. Bersanelli, M., et al., *Symptomatic COVID-19 in advanced-cancer patients treated with immune-checkpoint inhibitors: prospective analysis from a multicentre observational trial by FICOG.* Ther Adv Med Oncol, 2020. **12**: p. 1758835920968463.
42. Oliveira, L.M.d.S., et al., *Prevalence of anti-SARS-CoV-2 antibodies in outpatients of a large public university hospital in Sao Paulo, Brazil.* Revista do Instituto de Medicina Tropical de São Paulo, 2020. **62**.
43. Kindgen-Milles, D., et al., *Prevalence of SARS-COV-2 positivity in 516 German intensive care and emergency physicians studied by seroprevalence of antibodies National Covid Survey Germany (NAT-COV-SURV).* Plos one, 2021. **16**(4): p. e0248813.
44. Massoudi, N. and B. Mohit, *A Case-Control Study of the 2019 Influenza Vaccine and Incidence of COVID-19 Among Healthcare Workers.* J Clin Immunol, 2021. **41**(2): p. 324-334.
45. Murillo-Zamora, E., et al., *Male gender and kidney illness are associated with an increased risk of severe laboratory-confirmed coronavirus disease.* BMC infectious diseases, 2020. **20**(1): p. 1-8.
46. Angulo-Zamudio, U.A., et al., *Analysis of Epidemiological and Clinical Characteristics of COVID-19 in Northwest Mexico and the Relationship Between the Influenza Vaccine and the Survival of Infected Patients.* Front Public Health, 2021. **9**: p. 570098.
47. Alamdari, N.M., et al., *Mortality risk factors among hospitalized COVID-19 patients in a major referral center in Iran.* The Tohoku journal of experimental medicine, 2020. **252**(1): p. 73-84.
48. Sanchez-Garcia, C., et al., *History of influenza immunization in COVID-19 patients: impact on mortality.* Gac Med Mex, 2021. **157**(1): p. 102-106.

49. Poblador-Plou, B., et al., *Baseline chronic comorbidity and mortality in laboratory-confirmed COVID-19 cases: results from the PRECOVID study in Spain.* International journal of environmental research and public health, 2020. **17**(14): p. 5171.
50. Sardinha, D.M., et al., *Analysis of 472,688 severe cases of COVID-19 in Brazil showed lower mortality in those vaccinated against influenza.* medRxiv, 2021.
51. Marín‐Hernández, D., R.E. Schwartz, and D.F. Nixon, *Epidemiological evidence for association between higher influenza vaccine uptake in the elderly and lower COVID‐19 deaths in Italy.* Journal of medical virology, 2021. **93**(1): p. 64.
52. Zanettini, C., et al., *Influenza Vaccination and COVID19 Mortality in the USA.* MedRxiv, 2020.
53. Cocco, P., et al., *Vaccination against seasonal influenza and socio-economic and environmental factors as determinants of the geographic variation of COVID-19 incidence and mortality in the Italian elderly.* Preventive Medicine, 2021. **143**: p. 106351.
54. Arokiaraj, M.C., *Considering interim interventions to control COVID-19 associated morbidity and Mortality—Perspectives.* Frontiers in public health, 2020. **8**: p. 444.
55. Amato, M., et al., *Relationship between influenza vaccination coverage rate and COVID-19 outbreak: an Italian ecological study.* Vaccines, 2020. **8**(3): p. 535.
